# Drivers of the reduction in childhood diarrhea mortality 1980-2015 and interventions to eliminate preventable diarrhea deaths by 2030

**DOI:** 10.7189/jogh.09.020801

**Published:** 2019-12

**Authors:** Robert Black, Olivier Fontaine, Laura Lamberti, Maharaj Bhan, Luis Huicho, Shams El Arifeen, Honorati Masanja, Christa Fischer Walker, Tigest Ketsela Mengestu, Luwei Pearson, Mark Young, Nosa Orobaton, Yue Chu, Bianca Jackson, Massee Bateman, Neff Walker, Michael Merson

**Affiliations:** 1 Johns Hopkins University, Bloomberg School of Public Health, Department of International Health, Institute for International Programs, Baltimore, Maryland, USA; 2 World Health Organization, Department of Maternal, Newborn, Child and Adolescent Health Child and Adolescent Health and Development, Geneva, Switzerland; 3 Bill & Melinda Gates Foundation, Enteric Diarrheal Diseases, Seattle, Washington, USA; 4 Indian Institute of Technology, New Delhi, India; 5 Centro de Investigación en Salud Materna e Infantil, Centro de Investigación para el Desarrollo Integral y Sostenible and School of Medicine, Lima, Peru; 6 International Centre for Diarrhoeal Disease Research, Bangladesh (icddr,b), Dhaka, Bangladesh; 7 Ifakara Health Institute, Ifakara, Tanzania; 8 US Centers for Disease Control and Prevention, Maternal and Child Health, Windhoek, Namibia; 9 World Health Organization, Mbabane, Swaziland; 10 United Nations Children's Fund (UNICEF), New York, New York, USA; 11 United Nations Children's Fund (UNICEF), New York, New York, USA; 12 Bill & Melinda Gates Foundation, Maternal, Newborn and Child Health, Seattle, Washington, USA; 13 Johns Hopkins University, Bloomberg School of Public Health, Department of International Health, Institute for International Programs, Baltimore, Maryland, USA; 14 Johns Hopkins University, Bloomberg School of Public Health, Department of International Health, Institute for International Programs, Baltimore, Maryland, USA; 15 US Agency for International Development (USAID), Jakarta, Indonesia; 16 Johns Hopkins University, Bloomberg School of Public Health, Department of International Health, Institute for International Programs, Baltimore, Maryland, USA (deceased); 17 Duke University, Duke Global Health Institute, Durham, North Carolina, USA

## Abstract

**Background:**

Childhood diarrhea deaths have declined more than 80% from 1980 to 2015, in spite of an increase in the number of children in low- and middle-income countries (LMIC). Possible drivers of this remarkable accomplishment can guide the further reduction of the half million annual child deaths from diarrhea that still occur.

**Methods:**

We used the Lives Saved Tool, which models effects on mortality due to changes in coverage of preventive or therapeutic interventions or risk factors, for 50 LMIC to determine the proximal drivers of the diarrhea mortality reduction.

**Results:**

Diarrhea treatment (oral rehydration solution [ORS], zinc, antibiotics for dysentery and management of persistent diarrhea) and use of rotavirus vaccine accounted for 49.7% of the diarrhea mortality reduction from 1980 to 2015. Improvements in nutrition (stunting, wasting, breastfeeding practices, vitamin A) accounted for 38.8% and improvements in water, sanitation and handwashing for 11.5%. The contribution of ORS was greater from 1980 to 2000 (58.0% of the reduction) than from 2000 to 2015 (30.7%); coverage of ORS increased from zero in 1980 to 29.5% in 2000 and more slowly to 44.1% by 2015. To eliminate the remaining childhood diarrhea deaths globally, all these interventions will be needed. Scaling up diarrhea treatment and rotavirus vaccine, to 90% coverage could reduce global child diarrhea mortality by 74.1% from 2015 levels by 2030. Adding improved nutrition could increase that to 89.1%. Finally, adding increased use of improved water sources, sanitation and handwashing could result in a 92.8% reduction from the 2015 level.

**Conclusions:**

Employing the interventions that have resulted in such a large reduction in diarrhea mortality in the last 35 years can virtually eliminate remaining childhood diarrhea deaths by 2030.

Child mortality gradually declines as countries improve their social, environmental and economic conditions [1]. Since the 1980s there has been a focus on accelerating that decline by implementing interventions to address the major causes of child death [2]. A global commitment to reduce child mortality by 2015 was made in the Millennium Development Goals (MDG) [3] and has been renewed in the Sustainable Development Goals (SDG) to be achieved by 2030 [4]. The latter has a target for all countries of an under-five mortality rate of no more than 25 per 1000 live births. For childhood diarrhea a target of fewer than 1 death per 1000 live births (compared to 6 per 1000 live births in 2015) has been the aspiration [5].

Diarrhea was recognized in the 1970s to be a major cause of child death in low- and middle-income countries (LMIC) [6]. This recognition and the demonstration that an oral rehydration solution (ORS) could prevent mortality through treatment of dehydrating diarrhea [7] led the World Health Organization (WHO) to launch a Programme for Control of Diarrhoeal Diseases in 1978 [8]. Subsequent changes in the leadership and approaches for control of childhood diarrhea at the global level are described in **Box 1**. In parallel, efforts to improve child nutrition and enhance water supply and sanitation have continued with varying levels of intensity.

Box 1 **Diarrheal diseases control programs**

Understanding the leadership and governance history of diarrheal diseases control programs and the reasons behind major program decisions is essential to determine future priorities. The WHO Programme for Control of Diarrheal Diseases (CDD) was launched in 1978, with an objective to reduce diarrhea-associated morbidity and mortality among infants and young children in LMIC [3]. CDD developed [9] a simple approach for the assessment and treatment of diarrheal disease and dehydration that underlines the guidelines used today. This case management approach included the use of ORS [10] and continued feeding, including breastfeeding, during diarrhea, [11] and limited the use of antibiotics to only cases of bloody diarrhea [12]. Continued feeding was a paradigm shift away from the practice of withholding foods during diarrhea and was shown to reduce malnutrition and mortality in infants and children. Additionally, CDD research helped improve ORS [13] and increase its stability, acceptability and efficacy [14,15]. In the late 1980s, with a reduction in hospitalizations and hospital mortality due to diarrhea clearly established, the CDD Program strengthened its activities on the management of diarrhea at home. In 1989, CDD identified a diarrhea syndrome termed persistent diarrhea, characterized as an episode of diarrhea continuing without interruption for 14 days or more [16]. This syndrome is associated with malnutrition and a higher case fatality than acute diarrhea [17]. Research led to the development of a treatment algorithm for this syndrome [18]. In 2004, WHO and UNICEF recommended zinc supplementation during diarrhea based on studies showing that zinc shortened the duration of acute diarrhea [19] and prevented subsequent episodes in the 2 to 3 months following its use [20].

The CDD Program emphasized clinical training for health workers and training in program planning, management, and evaluation for senior and mid-level program managers. After operating as a stand-alone program for more than 10 years, the Program was combined with the Acute Respiratory Infection Control Program in 1990. In 1997 this combined program was expanded with the launch by WHO and UNICEF of the Integrated Management of Childhood Illness (IMCI) approach to address more comprehensively the main causes of under-five mortality: diarrhea, pneumonia, malaria, measles, and malnutrition. IMCI focused on improving 1) health worker skills; 2) health systems; and 3) family and community practices. WHO took responsibility for the first two components and UNICEF for the third component, which later expanded to include integrated community case management of diarrhea, pneumonia and malaria by trained community health workers. While this approach had some operational advantages, the types of support to countries provided by the CDD program which were responsible for much of its success, including managerial training, external program reviews, and national surveys of ORS access and use, were curtailed.

Still being implemented today, IMCI has undergone a number of evaluations and reviews [21-24]. A multi-country evaluation of IMCI in the 1990s found that countries struggled to scale up IMCI with sufficient quality and that most governments failed to provide IMCI a designated budget as they considered it a clinical strategy rather than an operational program [21]. A WHO 2016 review of IMCI identified several important deficiencies that limited the potential impact of the approach. These included: fragmented global strategies, lack of evidence on effectiveness of all components, poor integration of delivery strategies into policy and programming; and lack of accountability by WHO and UNICEF for IMCI globally and in countries. Some felt that IMCI’s focus on specific diseases and on facilities had detracted from a holistic view of child health. Nevertheless, IMCI ushered in a transformation in how the global public health community prioritized child health services.

Even with the progress on child survival there were still more than five million deaths in children less than five years of age, including a half million diarrhea deaths in 2015 [25]. We sought to review the progress with diarrheal disease control efforts along with other influences on diarrhea mortality to determine what were the possible drivers of the decline in diarrhea mortality from 1980 to 2015. Understanding these drivers may be useful to achieve the goal of ending preventable childhood diarrhea deaths by 2030 [5].

## METHODS

Mortality rates and numbers of deaths for 1990-2015 for children under five years of age and for the neonatal period were obtained from UN Interagency Group on Mortality Estimation (IGME) [26]. Under-five deaths and under-five mortality rates (U5MR) were based on data by five-years intervals available from the UN Population Division for 1960-1990 and interpolated assuming constant rate of change for years in between [27]. All-cause mortality proportions for neonates and children aged 1-59 months for the 1960-1989 were estimated using the neonatal/under-five ratio back extrapolated based on the within-country trend of U5MR from IGME data 1990-2015.

Global and national mortality from diarrhea in children under five years of age was estimated along with other causes by the World Health Organization for 2000-2015 [25]. Data from vital registration were used directly when available, otherwise vital registration and verbal autopsy data were utilized in multinomial logistic regression models to determine the distribution of the causes of death. For estimates of the number of deaths and rate of mortality due to diarrhea for years prior to 2000 cause-specific diarrhea fractions from vital registration and verbal autopsy data were used as input data to train a single-cause quadratic models with U5MR, source of input (verbal autopsy vs vital registration) and region as covariates. Models were fitted for neonatal and 1-59 month-olds’ deaths separately. The proportions of deaths in neonates and in 1-59 month-olds due to diarrhea were multiplied by the numbers of total deaths within each age group to derive the number of diarrhea deaths. For estimates of the causes of death other than diarrhea for 1980, the 2000-2015 country-level cause-specific death fractions were used as input data to train the multi-cause model with U5MR, endemic malaria and region as covariates, with models fitted for neonatal and 1-59 month-olds’ deaths separately. We used diarrhea fraction as estimated above and used AIDS deaths estimates from UNAIDS [28] assuming all to be after the neonatal period, and normalized the fraction for the rest of causes so they would add up to one. We calculated diarrhea-specific mortality rates (DSMR, diarrhea deaths per 1000 live births) for children under five years of age using the number of births from the UN Population Division [27].

The Lives Saved Tool (*LiST*) was used to estimate the possible drivers of the reduction in childhood diarrhea mortality from 1980 to 2015 [29]. The general approach used in *LiST* is that interventions have an estimated efficacy in reducing cause-specific mortality or levels of risk factors. As intervention coverage increases cause-specific mortality will decrease based on the magnitude of changes and the efficacy of the interventions. For risk factors, such as stunting or wasting, their relative risk or odds ratio for cause-specific mortality are included and as their prevalence decreases, mortality decreases. *LiST* models the effects of changes in preventive interventions and in risk factors first and therapeutic interventions act on the residual diarrheal cases that are not prevented.

In *LiST*, the causal pathways between interventions, risk factors (including behaviors) and diarrhea mortality are complex (Figure S1 and Figure S2 in **Online Supplementary Document**). Some interventions are specifically targeted to diarrhea including treatment of diarrhea or prevention due to rotavirus vaccine, while others have more indirect effects via nutritional status and interventions that prevent diarrhea or reduce the risk of fatality. The various linkages in *LiST* related to diarrhea mortality as well as the efficacy and risk values used in the model are published [29] or on the *LiST* website (www.livessavedtool.org). We assessed the impact of interventions individually and grouped as 1) direct diarrhea interventions (treatment with ORS, treatment with zinc, antibiotics for dysentery, treatment of persistent diarrhea, rotavirus vaccine), 2) nutrition risk factors and interventions (stunting, wasting, early initiation of breastfeeding, breastfeeding practices and vitamin A supplementation) and 3) water, sanitation, and hygiene interventions (WASH) (improved sanitation and water source together and handwashing). We used *LiST* to estimate which interventions or risk factors were responsible for the reduction in DSMR. We did this comparing 2015 to 1980, as well as for two sub-periods, comparing 2000 to 1980 and 2015 to 2000.

Data on coverage of interventions and prevalence of risk factors and behaviors, as well as mortality rates and causes, were available for 50 LMIC. Data sources are described in Table S1 in **Online Supplementary Document** and the values for 1980, 2000, and 2015 for the 50 countries in Table S2 in **Online Supplementary Document**. Together these 50 countries accounted for an estimated 9 040 363 deaths in children under five in 1980, representing 65% of all child deaths that year (13 928 041) [30]. By 2015, these countries accounted for an estimated 78% of global child deaths (4 612 919 of 5 944 556) [26].

In addition to the retrospective analyses, the potential impact of scaling up different packages of interventions to universal coverage (90%) by 2030 was explored in three different scenarios (Table S3 in **Online Supplementary Document**).

Scenario 1: Direct diarrhea interventions
Scenario 2: Direct diarrhea interventions and nutrition
Scenario 3: Direct diarrhea interventions, nutrition and WASH

National-level projections in *LiST* started using data from 2015 and interventions were scaled up linearly to 90% by 2030. For scenarios two and three, the impact of meeting World Health Assembly targets for reductions in number of children stunted (40% reduction in number of children stunted) and prevalence of wasting (<5% prevalence of wasting) were used. Because *LiST* cannot model targets of numbers of children stunted, the target of 50% reduction in prevalence of stunting was used as an approximation, accounting for anticipated population growth and a corresponding likely increase in the number of children stunted without additional intervention.

## RESULTS

### Trends in child mortality due to diarrheal disease

**Figure 1:**
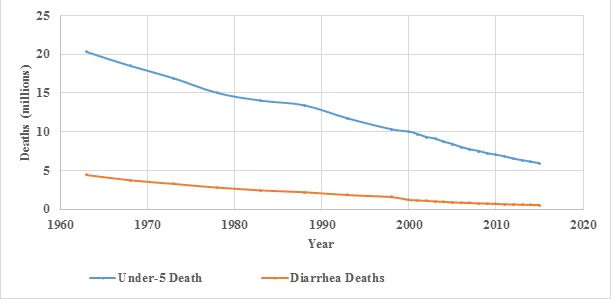
Global number of all-cause deaths and diarrhea deaths among children under-five, 1960-2010.

There was a large decline in the numbers of all-cause and diarrhea deaths in children under five years of age from 1980-2015 (**Figure 1**). In 1980 it is estimated that there were 2.7 million (95% Uncertainty Interval (UI) (2.2-3.1 million) under-five deaths from diarrhea, accounting for 18.2% of all under-five deaths and 26.1% of deaths in the 1-59 month-olds. In 2015 the number of diarrhea deaths was estimated to be 0.5 million (UI 0.4-0.7 million) and the fraction of all deaths due to diarrhea was reduced to 9.1% among under-five year olds and 15.9% among 1-59 month-olds. This decline in the number of diarrhea deaths occurred in a period when the number of children in the world increased by 27.2% [31].

The numbers of diarrhea deaths had been very high in some South Asia (SA) and sub-Saharan Africa (SSA) countries albeit reduced in the 35-year period (Table S4 in **Online Supplementary Document**). Together, SA and SSA accounted for 71.8% of diarrhea deaths in 1980 increasing to 90.6% in 2015. The relative contribution of SA vs SSA to global mortality also changed during this period. Among the top ten countries with the highest number of diarrhea deaths, three were from SA and two were from SSA in 1980 (Table S4 in **Online Supplementary Document**). In contrast, in 2015, three SA countries were among top ten, and the other seven countries were in SSA.

**Figure 2:**
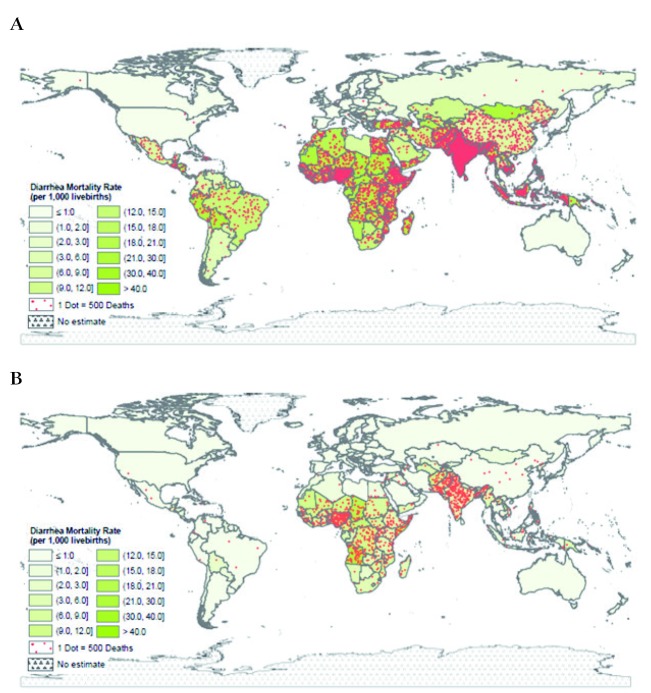
Geographic distribution of diarrhea deaths and mortality. **Panel A.** Geographic distribution of diarrhea deaths and mortality in 1980. **Panel B.** Geographic distribution of diarrhea deaths and mortality in 2015.

The diarrhea-specific mortality rate also shows a large decline, being 21.3 and 5.7 diarrhea deaths per 1000 live births in 1980 and 2015, respectively (Figure S3 in **Online Supplementary Document**). The diarrhea mortality rates varied by country in 1980 and 2015 with the highest rates in SA and SSA (**Figure 2**, Panels A and B).

### Drivers of diarrhea mortality reduction in LMIC 1980-2015

We compared the estimated change in DSMR from *LiST* to the national estimates of diarrhea mortality decline described above for the 50 countries. *LiST* estimated a 55.5% reduction in DSMR compared to a 79.9% actual reduction in mortality, apparently underestimating the overall decline by one third (Table S5 in **Online Supplementary Document**) [25].

**Figure 3:**
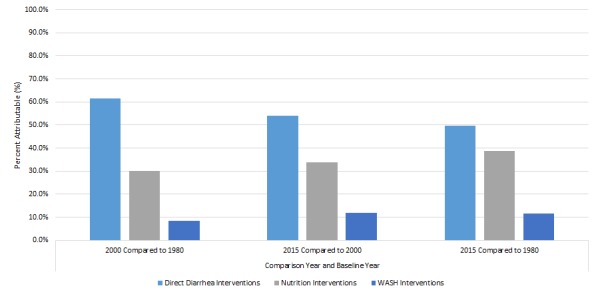
Attribution of mortality reduction to intervention types.

The largest percentage of the DSMR reduction for a single factor comparing 2015 to 1980 was attributed to use of ORS (36.2%) (**Table 1**). Overall, the five direct diarrhea interventions accounted for 49.7% of the reduction. Nutrition factors accounted for 38.8% of the reduction, predominantly due to reduction in stunting and vitamin A supplementation. WASH interventions accounted for 11.5%. The attribution of ORS for mortality reduction was higher (58.0%) for 1980 to 2000 than for 2000 to 2015 (30.7%). The latter time period had a higher attribution of mortality reduction to decreased stunting (5.5% vs 15.9%). It also had attributions to treatment for persistent diarrhea (12.6%), rotavirus vaccine (5.4%) and zinc for diarrhea treatment (4.9%), as these were introduced during this period. Overall, direct diarrhea interventions had a larger attribution in the first time period; attribution to nutrition and WASH interventions was somewhat greater in the second period (**Figure 3**).

**Table 1:** Changes in coverage of interventions or prevalence of risk factors and attribution of diarrhea deaths reduced 1980 to 2015

Intervention/Risk factor	Coverage/Prevalence weighted by national live births (%)			Attribution of change in diarrhea deaths (%)		
	**1980**	**2000**	**2015**	**2000 compared to 1980**	**2015 compared to 2000**	**2015 compared to 1980**
Oral rehydration solution*	0.0	29.5	44.1	58.0	30.7	36.2
Global stunting (<-2 SD) rate†	57.3	48.7	36.5	5.5	15.9	19.5
Vitamin A supplementation†	0	26.1	68.5	9.5	17.9	16.0
Persistent diarrhea treatment*	0.0	0.0	33.0	0.0	12.6	6.6
Improved sanitation + improved water source‡	27.4	30.1	41.6	2.9	8.7	6.5
Handwashing with soap‡	8.6	13.9	18.3	5.5	3.2	5.0
Age appropriate breastfeeding§,†	35.8	37.2	40.6	9.2	0.0	3.1
Zinc for diarrhea treatment*	0.0	2.4	12.0	1.2	4.9	2.9
Rotavirus vaccine*	0.0	0.0	25.5	0.0	5.4	2.7
Antibiotics for dysentery*	12.3	18.7	21.1	2.3	0.5	1.3
Early initiation of breastfeeding†	26.0	37.5	58.0	0.2	0.1	0.2
Global wasting (<-2 SD) rate‖†	15.0	12.9	13.3	5.8	0.0	0.0

*Direct diarrhea interventions.

†Nutrition interventions.

‡Water, sanitation and hygiene interventions.

§Exclusive breastfeeding 1-5 months (for prevalence of other breastfeeding practices see Table S2 in **Online Supplementary Document**). While prevalence of exclusive breastfeeding in this age group increased, the percentage of children not breastfed in all age groups increased between the years 1980 and 2015.

‖While prevalence of moderate + severe wasting (<-2DS) decreased over the time period, the prevalence of severe wasting (<-3SD) increased (Table S2 in **Online Supplementary Document**).

### Potential impact on diarrhea mortality by 2030

We assessed the potential impact by 2030 of three scenarios for scaling up interventions from 2015 to 2030:

Scenario 1: In this scenario, we assessed 90% coverage of direct diarrhea interventions first in *LiST* because these are considered highly cost-effective and more feasible to scale up in the short term than interventions for nutrition and WASH. These five direct interventions could result in a 74.1% reduction in DSMR (Table S6 in **Online Supplementary Document**). Of this reduction coverage of ORS treatment was the major driver accounting for 51.6%. In addition, zinc treatment for diarrhea (16.9%), persistent diarrhea (12.1%), rotavirus vaccine (12.0%) and antibiotics for dysentery (7.3%) all contributed (Table S7 in **Online Supplementary Document**). Fifteen countries, in which the DSMR was relatively low in 2015, would be able to reduce their DSMR to <1 diarrhea deaths per 1000 live births in this scenario (Table S8 and Figure S4 in **Online Supplementary Document**).

Scenario 2: If improved nutrition is included in addition to achieving universal coverage of direct diarrhea interventions, the DSMR for these countries could be reduced by an estimated 89.1% (Table S6 in **Online Supplementary Document**). In this scenario, the reduction is attributable primarily to increases in ORS use (22.8%), reduction in stunting prevalence (21.6%), improved breastfeeding practices (17.3%) and reduced wasting (10.1%) (Table S7 in **Online Supplementary Document**). The attribution to the diarrhea treatment interventions is substantially reduced in this scenario because the preventive interventions are included first in *LiST* and the treatment interventions act on the residual cases. Meeting these additional targets is estimated to reduce the DSMR of an additional 22 countries to <1 diarrhea death per 1000 live births (Table S8 and Figure S4 in **Online Supplementary Document**).

Scenario 3: If 90% coverage of WASH interventions is also achieved, diarrhea mortality could be reduced by 92.8% (Table S6 in **Online Supplementary Document**) and to less than 1 diarrhea death per 1000 live births in all of the countries included in this analysis, except for Angola and Sierra Leone (Table S8 and Figure S4 in **Online Supplementary Document**). In this scenario changes in stunting prevalence (17.1%), handwashing with soap (15.7%), followed by ORS coverage (14.4%) and changes in breastfeeding practices (13.5%) and improved sanitation and water source (13.1%) are the predominant drivers (Table S7 in **Online Supplementary Document**).

## DISCUSSION

Childhood diarrhea mortality has declined substantially since 1980. Our estimates of the number of deaths due to diarrhea among children under-five compare favorably with previous reviews. Snyder and Merson [6] reported in 1982 an estimate of 4.6 million diarrhea deaths. The average year of data collection for the publications included in their analysis was 1967. From our trend of diarrhea deaths, we estimate that in 1967 there were 3.9 million (Interquartile Range (IQR) = 3.6-4.2) diarrhea deaths, representing 20.6% of under-five deaths. Bern et al. estimated that there were 3.3 (IQR = 1.5-5.1) million diarrhea deaths based on studies with an average data collection year of 1980 [32]. Our estimate of 2.7 million (IQR = 2.5-2.8) diarrhea deaths in 1980 was lower, but the IQR of Bern and our analysis overlap. Kosek et al. reported that there were 2.5 million (IQR = 2.1-4.7) under-five diarrhea deaths globally based on studies with an average data collection year of 1990 [33]. Our estimate of 2.0 million (IQR = 1.9-2.1) in 1990 and this estimate also have overlapping IQR. Our estimate of child diarrhea deaths for 2015 of 526 000 (95% CI = 418 000-691 000) is slightly higher than the 485 800 estimate by the Global Burden of Diseases (GBD) [25,34].

Our results confirm that improving access to and use of ORS for dehydrating diarrhea had a very substantial effect on reducing diarrheal mortality, especially from 1980 to 2000 when WHO, UNICEF and bilateral donors supported national diarrheal disease control programs. In the MDG era since 2000, integrated health facility approaches to management of child illness have not resulted in large improvements in diarrhea treatment, with currently only two in five episodes receiving ORS [35]. Programs to provide community-based treatment of diarrhea and other life-threatening diseases where implemented have resulted in greater access and coverage [36]. In spite of the proven benefits and a global recommendation made in 2004 by WHO and UNICEF, coverage with therapeutic zinc remains very low except in a few countries [37]. Rotavirus vaccine implementation has accelerated in recent years and coverage is high in countries that have received support from GAVI, but the benefit is constrained by the relatively low efficacy in LMIC with high child mortality [38].

Reductions in stunting and micronutrient deficiencies of children that have occurred in this 35-year period are undoubtedly related to socioeconomic changes [39]. Global poverty was estimated to be 41.9% in 1981 and 10.7% in 2013 and education, especially for girls, has increased substantially [40]. In addition, vitamin A supplementation has reduced child mortality and the current high coverage needs to be sustained in remaining high mortality settings [41]. In our analysis WASH improvements had less effect on diarrhea mortality than diarrhea treatment or reduction in nutritional risk factors in spite of large investments in improved water sources and sanitation for several decades. This may reflect limitations of the level of currently provided infrastructure and difficulties in changing behaviors.

A recent GBD publication on diarrhea included a decomposition analysis of the effect of 10 “risk factors” on childhood diarrhea mortality between 2000 and 2016 [34]. The factor with the largest attribution (11.8%) was childhood wasting followed by changes in sanitation (about 10%); improved sanitation and water source together was about 15%. In our *LiST* analysis from 2000 to 2015 none of the change in diarrhea mortality was attributed to wasting; in the 50 countries we analyzed the prevalence of moderate wasting declined slightly, but severe wasting increased. In our analysis, improved sanitation and water source were considered together because the best evidence for the effect on diarrhea came from recent trials that implemented both concurrently; we found a 6.5% attribution for the reduction of diarrhea deaths. The results of WASH intervention trials that we used had less effect than would be assumed from risk factor analyses which are based on observational data [42]. It is possible that confounding in observational studies may result in overestimation of the relationship of WASH characteristics to risk of diarrhea mortality. That childhood diarrhea incidence in LMIC in recent decades has not declined substantially [33,43] is consistent with findings of limited effects of WASH. The GBD analysis for 2000-2016 found a much lower attribution for ORS (about 7%) than in our analysis (31%). Although our attribution in 2000-2015 was less than in 1980-2000 there was an increase in ORS coverage in the 50 countries in the later period (29.5% to 44.1%), resulting in a substantial effect on mortality. It is not clear from the limited methods provided by GBD how to explain this difference, but since both analyses use the same source for the effectiveness of ORS [14] it must relate to either ORS coverage data or analytical methods.

Our analyses of the drivers of the decline in childhood diarrhea mortality were for 50 LMIC that had 65% of diarrhea deaths in 1980 and 78% in 2015 based on availability of data needed for the *LiST* analysis. We were able to attribute two thirds of the decline in childhood diarrhea mortality to changes in specific interventions or risk factors. Because we based the endpoint for changes in the drivers on the latest nationally representative survey data, we likely underestimated the attribution for countries when the survey was up five years before 2015. Limitations regarding data on intervention coverage decrease the certainty of the attributions and other possible drivers could not be included because of lack of data.

Existing proven interventions targeting vulnerable children are sufficient to achieve the goal of near elimination of child diarrhea deaths. The direct preventive and therapeutic interventions for diarrhea could achieve three-quarters of that goal [44,45] with most of that coming from higher coverage with ORS. Improvements in nutrition and WASH would contribute to further in diarrhea mortality and have other health and economic benefits. At global and national levels, successful implementation of these interventions is going to require effective and equitable coverage of interventions.

## Additional material

Online Supplementary Document

## References

[1] Hill K Pebley AR Child Mortality in the Developing World. Popul Dev Rev. 1989;15:657-87. 10.2307/1972594

[2] Bellagio Study Group on Child Survival. Knowledge into action for child survival. 2003;362:323-7. 10.1016/s0140-6736(03)13977-3 12892965

[3] United Nations. Millennium development goals report 2015. New York, NY. United Nations. 2015. Available: http://www.un.org/millenniumgoals/2015_MDG_Report/pdf/MDG%202015%20rev%20(July%201).pdf. Accessed: 25 April 2017.

[4] United Nations. Department of Economic and Social Affairs. Transforming our world: the 2030 agenda for sustainable development. Resolution adopted by the General Assembly 2015. Available: https://sustainabledevelopment.un.org/post2015/transformingourworld. Accessed: 25 April 2017.

[5] Chopra M Mason E Borrazzo J Campbell H Rudan I Liu L Ending of preventable deaths from pneumonia and diarrhoea: an achievable goal. Lancet. 2013;381:1499-506. 10.1016/S0140-6736(13)60319-0 23582721

[6] Snyder JD Merson MH The magnitude of the global problem of acute diarrhoeal disease: a review of active surveillance data. Bull World Health Organ. 1982;60:605. 6982783 PMC2536091

[7] Santosham M Greenough WB III Oral rehydration therapy: a global perspective. J Pediatr. 1991;118:S44-51. 10.1016/S0022-3476(05)81425-8 2007956

[8] Victora CG Bryce J Fontaine O Monasch R Reducing deaths from diarrhoea through oral rehydration therapy. Bull World Health Organ. 2000;78:1246-55. 11100619 PMC2560623

[9] World Health Organization. Programme for the Control of Diarrheal Diseases. The treatment of diarrhea: A manual for physicians and other senior health workers. Geneva, Switzerland: WHO Press, 1980.

[10] Mahalanabis D Choudhuri A Bagchi N Bhattacharya A Simpson T Oral fluid therapy of cholera among Bangladesh refugees. Johns Hopkins Med J. 1973;132:197-205. 4698667

[11] Chung AW The effect of oral feeding at different levels on the absorption of foodstuffs in infantile diarrhea. J Pediatr. 1948;33:1-13. 10.1016/S0022-3476(48)80147-2 18868991

[12] Haltalin KC Nelson JD Ring R III Sladoje M Hinton LV Double-blind treatment study of shigellosis comparing ampicillin, sulfadiazine, and placebo. J Pediatr. 1967;70:970-81. 10.1016/S0022-3476(67)80275-0 5338090

[13] Hoffman SL Moechtar MA Simanjuntak CH Punjabi NH Kumala S Sutoto Rehydration and maintenance therapy of cholera patients in Jakarta: citrate-based versus bicarbonate-based oral rehydration salt solution. J Infect Dis. 1985;152:1159-65. 10.1093/infdis/152.6.1159 3905981

[14] Munos MK Walker CLF Black RE The effect of oral rehydration solution and recommended home fluids on diarrhoea mortality. Int J Epidemiol. 2010;39:i75-87. 10.1093/ije/dyq025 20348131 PMC2845864

[15] Black RE The prophylaxis and therapy of secretory diarrhea. Med Clin North Am. 1982;66:611-21. 10.1016/S0025-7125(16)31410-9 7043125

[16] Black RE Persistent diarrhea in children of developing countries. Pediatr Infect Dis J. 1993;12:751-61, discussion 62-4. 10.1097/00006454-199309000-00010 8414804

[17] Bhandari N Bhan M Sazawal S Mortality associated with acute watery diarrhea, dysentery and persistent diarrhea in rural north India. Acta Paediatr. 1992;81:3-6. 10.1111/j.1651-2227.1992.tb12363.x 12286021

[18] World Health Organization. The treatment of diarrhoea: a manual for physicians and other senior health workers. 4th ed. Geneva, Switzerland: WHO Press; 2005.

[19] Sazawal S Black RE Bhan MK Bhandari N Sinha A Jalla S Zinc supplementation in young children with acute diarrhea in India. N Engl J Med. 1995;333:839-44. 10.1056/NEJM199509283331304 7651474

[20] Bhutta ZA Black RE Brown KH Gardner JM Gore S Hidayat A Prevention of diarrhea and pneumonia by zinc supplementation in children in developing countries: pooled analysis of randomized controlled trials. Zinc Investigators’ Collaborative Group. J Pediatr. 1999;135:689-97. 10.1016/S0022-3476(99)70086-7 10586170

[21] Bryce J Victora CG Habicht JP Vaughan JP Black RE The multi-country evaluation of the integrated management of childhood illness strategy: lessons for the evaluation of public health interventions. Am J Public Health. 2004;94:406-15. 10.2105/AJPH.94.3.406 14998804 PMC1448266

[22] Gera T Shah D Garner P Richardson M Sachdev HS Integrated management of childhood illness (IMCI) strategy for children under five. Cochrane Database Syst Rev. 2016;CD010123. 10.1002/14651858.CD010123.pub2 27378094 PMC4943011

[23] World Health Organization. The analytic review of the integrated management of childhood illness strategy. Final Report. 2003. Available: http://apps.who.int/iris/bitstream/10665/42965/1/9241591730.pdf. Accessed: 1 June 2017.

[24] Costello AM. Dalglish SL on behalf of the Strategic Review Study Team. Towards a Grand Convergence for child survival and health: A strategic review of options for the future building on lessons learnt from IMNCI. Geneva, Switzerland: WHO Press; 2016.

[25] Liu L Oza S Hogan D Chu Y Perin J Zhu J Global, regional, and national causes of under-5 mortality in 2000-15: an updated systematic analysis with implications for the Sustainable Development Goals. Lancet. 2016;388:3027-35. 10.1016/S0140-6736(16)31593-8 27839855 PMC5161777

[26] You D, Hug L, Ejdemyr S, Beise J. Levels and trends in child mortality. Report 2015. Estimates developed by the UN Inter-agency Group for Child Mortality Estimation. Available: https://www.unicef.org/publications/files/Child_Mortality_Report_2015_Web_9_Sept_15.pdf. Accessed 26 April 2017.

[27] United Nations, Department of Economic and Social Affairs, Population Division. World population prospects. Population indicators. 2017. Available: https://esa.un.org/unpd/wpp/Download/Standard/Population/. Accessed: 7 July 2017.

[28] Stover J. AIM: A Computer Program for Making HIV/AIDS Projections and Examining the Demographic and Social Impacts of AIDS. 2009. Available: http://data.unaids.org/pub/manual/2009/20090414_aim_manual_2009_en.pdf. Accessed: 25 April 2017.

[29] Fischer Walker CL Walker N The Lives Saved Tool (LiST) as a model for diarrhea mortality reduction. BMC Med. 2014;12:70. 10.1186/1741-7015-12-70 24779400 PMC4234397

[30] Roser M. Child Mortality. 2017. [cited 2017 Apr 26]. Available from: http://ourworldindata.org/child-mortality.

[31] United Nations. Department of Economic and Social Affairs. World economic situation and prospects as of mid-2017. 2017. Available: https://www.un.org/development/desa/dpad/document_gem/global-economic-monitoring-unit/world-economic-situation-and-prospects-wesp-report/. Accessed: 24 September 2017.

[32] Bern C Martines J de Zoysa I Glass RI The magnitude of the global problem of diarrhoeal disease: a ten-year update. Bull World Health Organ. 1992;70:705-14. 1486666 PMC2393403

[33] Kosek M Bern C Guerrant RL The global burden of diarrhoeal disease, as estimated from studies published between 1992 and 2000. Bull World Health Organ. 2003;81:197-204. 12764516 PMC2572419

[34] GBD Diarrhoeal Disease Collaborators Estimates of the global, regional, and national morbidity, mortality, and aetiologies of diarrhoea in 195 countries: a systematic analysis for the Global Burden of Disease Study 2016. Lancet Infect Dis. 2018;18:1211-28. 10.1016/S1473-3099(18)30362-1 30243583 PMC6202444

[35] Requejo JH Bryce J Barros AJ Berman P Bhutta Z Chopra M Countdown to 2015 and beyond: fulfilling the health agenda for women and children. Lancet. 2015;385:466-76. 10.1016/S0140-6736(14)60925-9 24990815 PMC7613194

[36] Young M Wolfheim C Marsh DR Hammamy D World Health Organization/United Nations Children’s Fund joint statement on integrated community case management: an equity-focused strategy to improve access to essential treatment services for children. Am J Trop Med Hyg. 2012;87:6-10. 10.4269/ajtmh.2012.12-0221 23136272 PMC3748523

[37] Tracking progress towards universal coverage for women’s, children’s and adolescents’ health. Countdown to 2030 The 2017 Report. Washington, DC: United Nations Children's Fund (UNICEF) and the World Health Organization (WHO), 2017.

[38] Lamberti LM Ashraf S Walker CLF Black RE A systematic review of the effect of rotavirus vaccination on diarrhea outcomes among children younger than 5 years. Pediatr Infect Dis J. 2016;35:992-8. 10.1097/INF.0000000000001232 27254030

[39] Ruel MT Alderman H Maternal, Child Nutrition Study G. Nutrition-sensitive interventions and programmes: how can they help to accelerate progress in improving maternal and child nutrition? Lancet. 2013;382:536-51. 10.1016/S0140-6736(13)60843-0 23746780

[40] The World Bank. IBRD-IDA. Poverty Data. 2017. Available: https://data.worldbank.org/topic/poverty. Accessed: 24 September 2017.

[41] Mayo-Wilson E Imdad A Herzer K Yakoob MY Bhutta ZA Vitamin A supplements for preventing mortality, illness, and blindness in children aged under 5: systematic review and meta-analysis. BMJ. 2011;343:d5094. 10.1136/bmj.d5094 21868478 PMC3162042

[42] Risk Factors Collaborators GBD Global, regional, and national comparative risk assessment of 84 behavioural, environmental and occupational, and metabolic risks or clusters of risks, 1990-2016: a systematic analysis for the Global Burden of Disease Study 2016. Lancet. 2017;390:1345-422. 10.1016/S0140-6736(17)32366-8 28919119 PMC5614451

[43] Fischer Walker CL Perin J Aryee MJ Boschi-Pinto C Black RE Diarrhea incidence in low-and middle-income countries in 1990 and 2010: a systematic review. BMC Public Health. 2012;12:220. 10.1186/1471-2458-12-220 22436130 PMC3323412

[44] World Health Organization Weekly Epidemiological Record - Relevé éptdôm hebd. 1989;68:313-20.

[45] Chopra M Mason E Borrazzo J Campbell H Rudan I Liu L Ending of preventable deaths from pneumonia and diarrhoea: an achievable goal. Lancet. 2013;381:1499-506. 10.1016/S0140-6736(13)60319-0 23582721

[46] Wolfheim C Fontaine O Merson MH Evolution of the World Health Organization’s programnatic actions for the control of diarrheal diseases. J Glob Health. 2019;9:020802 10.7189/jogh.09.020802 PMC6816052 31673346

