# Supplementary material for: Drivers of the reduction in childhood diarrhea mortality 1980-2015 and interventions to eliminate preventable diarrhea deaths by 2030
Source: J Glob Health. 2019 Oct 22;9(2):020801. doi: 10.7189/jogh.09.020801 (PMC6815873; doi:10.7189/jogh.09.020801)
Supplement: Online Supplementary Document [file jogh-09-020801-s001.pdf]

Web Appendix

Determinants of the reduction in Childhood Diarrhea Mortality 1980-2015 and Interventions to Eliminate Preventable Diarrhea Deaths by 2030

Robert Black, Olivier Fontaine, Laura Lamberti, Maharaj Bhan, Luis Huicho, Shams El Arifeen, Honorati Masanja, Christa Fischer Walker, Tigest Ketsela Mengestu, Luwei Pearson, Mark Young, Nosa Orobaton, Yue Chu, Bianca Jackson, Massee Bateman, Neff Walker, Michael Merson

Table of Contents

Webappendix Figure 1: The causal pathways between interventions and risk factors and neonatal mortality, as modeled in *LiST*......2

Webappendix Figure 2: The causal pathways between interventions and risk factors and post-neonatal child mortality, as modeled in *LiST*......3

Webappendix Figure 3: Global trend of under five mortality and diarrhea-specific mortality rates, 1960s-2010s......4

Webappendix Figure 4: Countries that would reduce diarrhea mortality to <1 death per 1000 live births in three scenarios 5

Webappendix Table 1: Data source documentation...... 6

Webappendix Table 2: Average coverage/prevalence of interventions in 50 countries included in analysis, weighted by number of live births in each country......7

Webappendix Table 3: Description of the interventions and risk factors included in each universal coverage scale-up scenario......8

Webappendix Table 4: Number of diarrhea deaths among children under-five for 87 LMIC in 1980 and 2015......9

Webappendix Table 5: Comparison of change in DSMR calculated from mortality trends versus trends that calculated from *LiST* projections in 50 LMIC......12

Webappendix Table 6: The impact on diarrhea-specific mortality (DSMR) if increased coverage of different packages of interventions is achieved by 2030......14

Webappendix Table 7: The percent of child lives saved in 2030 attributable to each intervention in the three scale-up scenarios......16

Webappendix Table 8: Countries that would reduce diarrhea mortality to <1 death per 1000 live births in three scenarios......17

Webappendix Figure 1

The causal pathways between interventions and risk factors and neonatal mortality, as modeled in *LiST*.

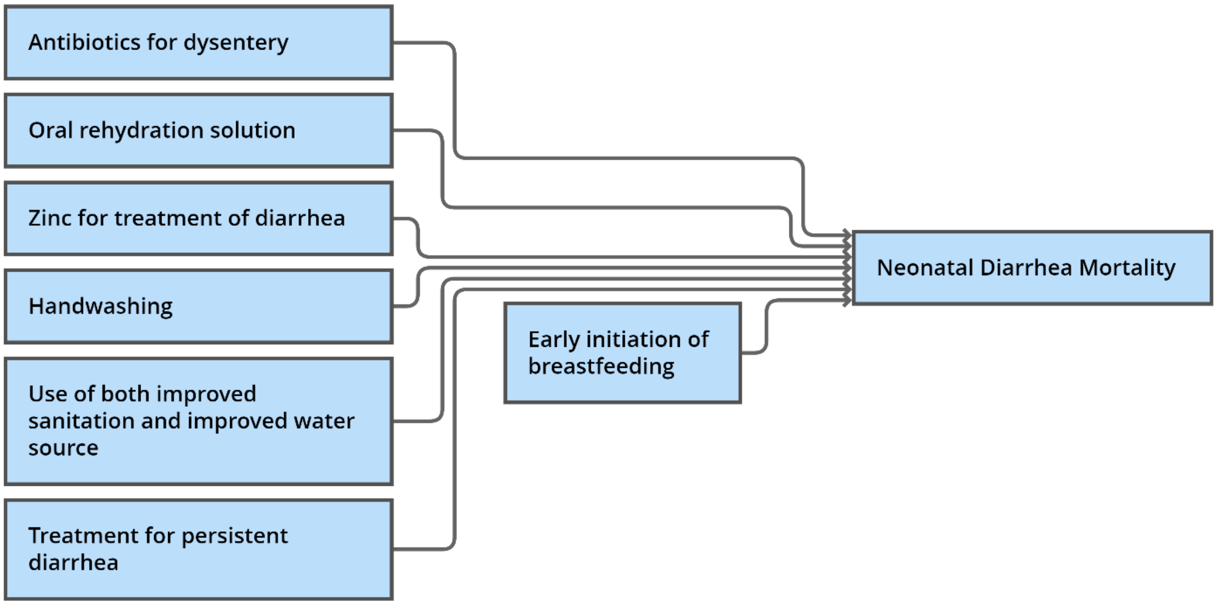

Webappendix Figure 2

The causal pathways between interventions and risk factors and post-neonatal child mortality, as modeled in *LiST.*

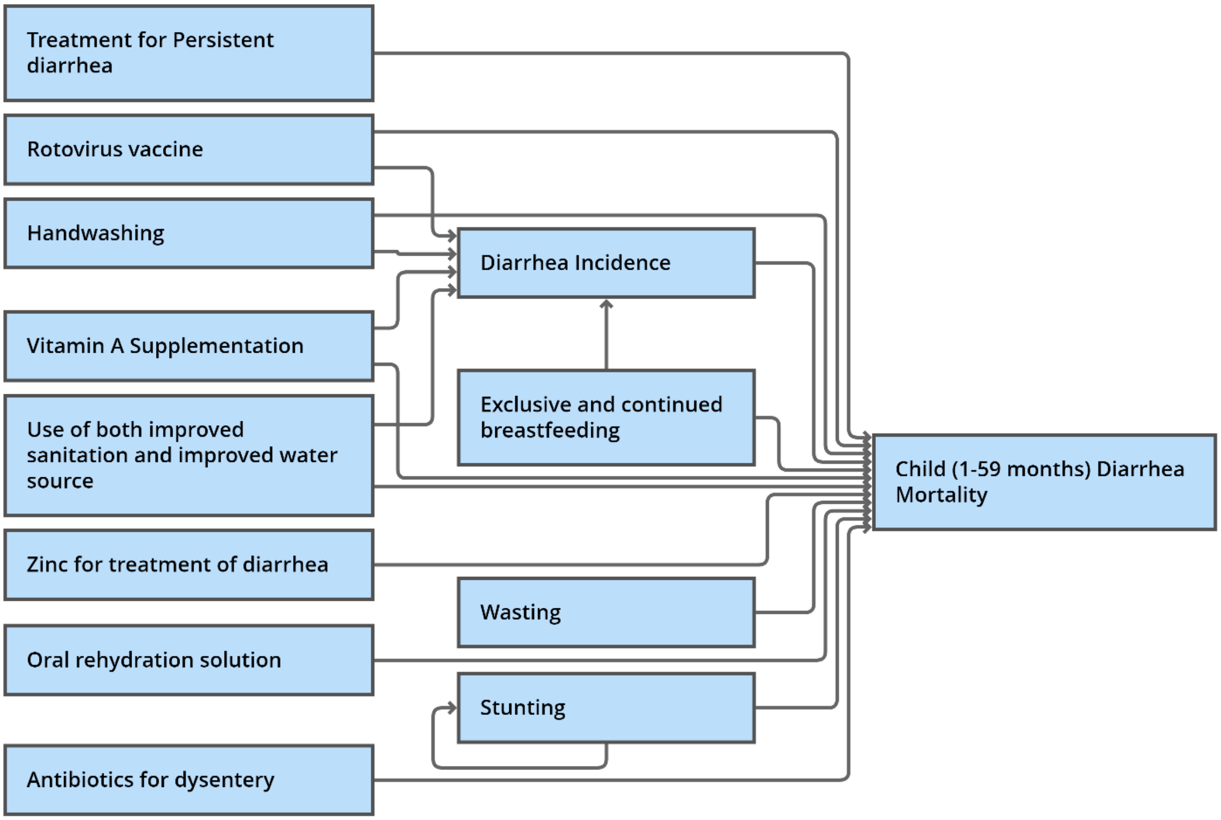

[1] The following interventions also have causal linkages to diarrhea mortality in children aged 1-59 months through risk factors, and can be modelled in LiST, but were not included in the present analyses because of a lack of coverage data: treatment for severe acute malnutrition (SAM), treatment for moderate acute malnutrition (MAM), balanced energy supplementation in pregnancy, intermittent preventive treatment of malaria in pregnancy (IPTp), insecticide-treated bed nets (ITN)/indoor residual spraying (IRS) for prevention of malaria, multiple micronutrient supplementation in pregnancy, complementary feeding, kangaroo mother care (KMC). For the full visualization of all causal linkages in LiST, including all interventions that have linkages to diarrhea mortality in children, please see http://listvisualizer.org/.

Webappendix Figure 3

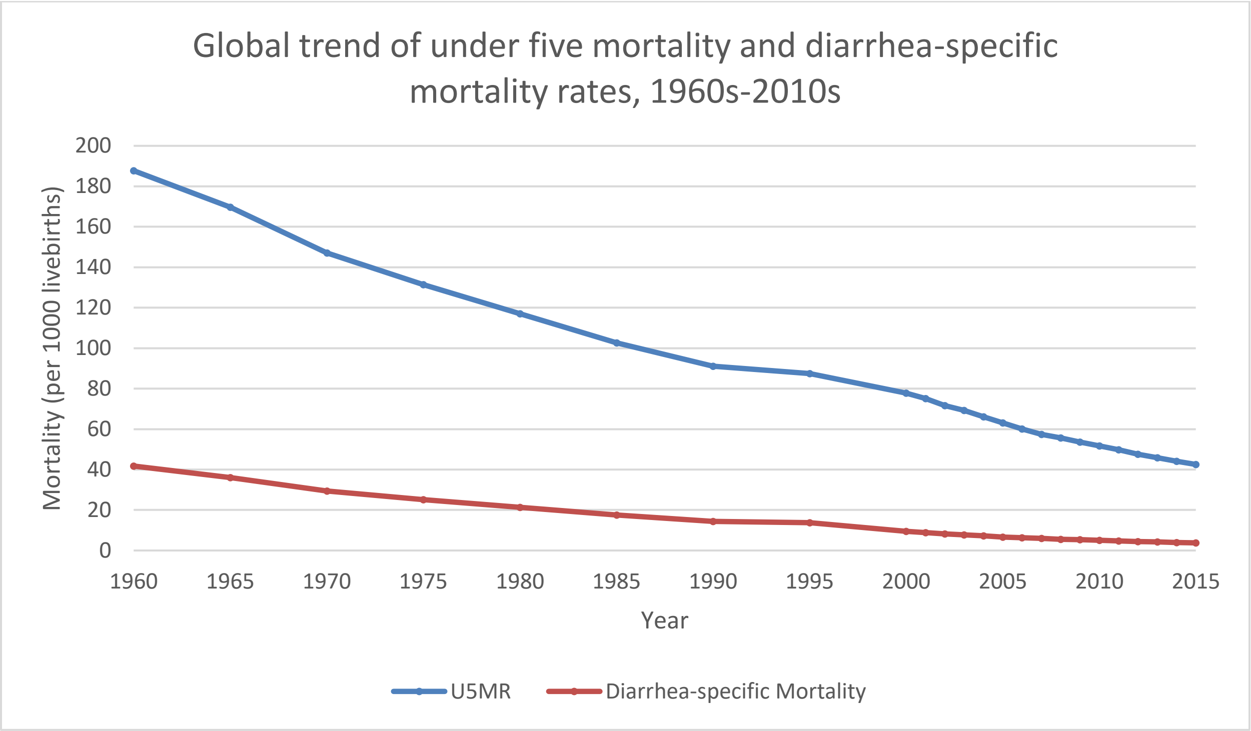

Webappendix Figure 4

Countries that would reduce diarrhea mortality to <1 death per 1000 live births in three scenarios

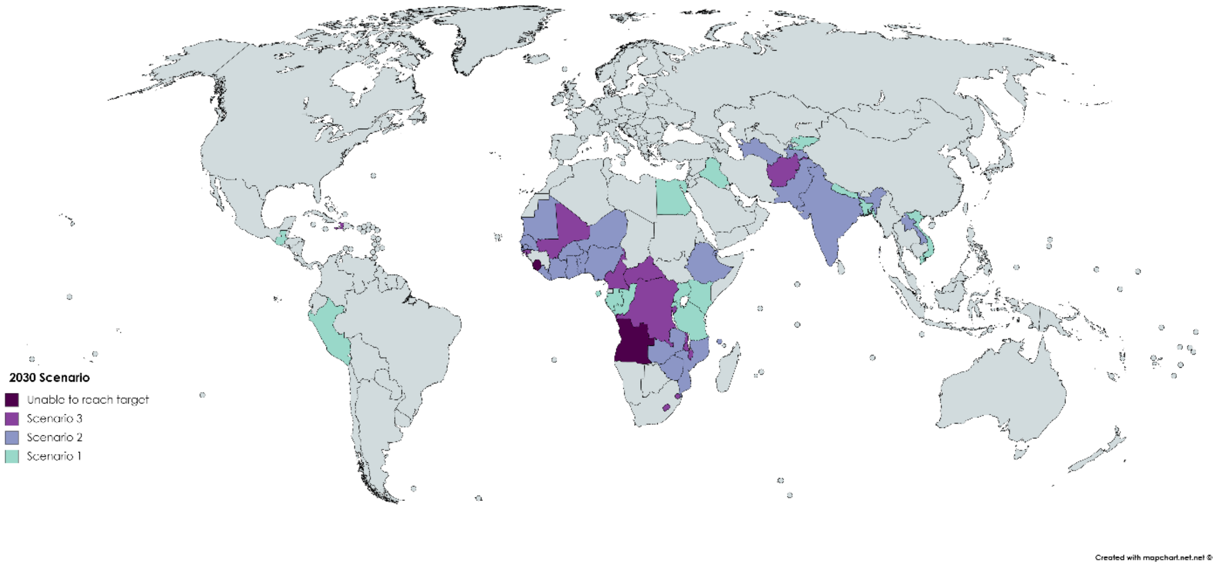

Webappendix Table 1

Data source documentation

Indicator	Source(s)	Assumptions
Neonatal, infant and under-five mortality rates	United Nations Population Division	None
Height-for-age Z score (HAZ) distribution data, segregated by age group	Overall stunting rates (% of children under five greater than -2 SD below reference population mean and % of children under five greater than -3SD below reference population mean) for year 1985[58]<br><br>When available, data from nationally representative surveys were used*	Modeling in LiST required Stunting distribution pattern by age. Matched stunting distribution by age from earliest DHS, or similar survey. Assumed 1980 stunting distribution equal to 1985 stunting distribution[58]
Weight-for-age Z score (WHZ) distribution data, segregated by age group	Nationally representative surveys*	Assumed 1980 coverage equal to earliest measured from nationally representative surveys. For subsequent years, linear changes were assumed between measured values from nationally representative surveys
Cause of death structure (i.e. percentage of deaths due to each major cause of death)	Maternal Child Epidemiology Estimation	None
Antibiotics for dysentery	Nationally representative surveys*	Coverage equal to 0% in year 1960and linear scale up until earliest measured data
Early Initiation of breastfeeding	Demographic and Health Surveys, Multiple Indicator Cluster Surveys, other national surveys	Assumed 1980 coverage equal to earliest measured
Hand washing with soap	Joint Monitoring Programme	Assumed coverage equal to coverage of water connection in the home in 1980 and linear scale up to earliest measured data from JMP or 2009 global estimate
Improved sanitation - Utilization of latrines or toilets + improved water source	Joint Monitoring Programme	Assumed coverage equal to 0% in year 1960 and linear scale up until earliest measured data. Effectiveness applies only when improved sanitation and improved water are both present.
Rotavirus vaccine (two doses)	UNICEF vaccine coverage data	Assumed 0% unless measured data available
Oral rehydration solution	Nationally representative surveys*	Assumed coverage equal to 0% in year 1980 and linear scale up until earliest measured data
Vitamin A supplementation	UNICEF Vitamin A supplementation data	Assumed overage equal to 0% in 1980. Assumed coverage equal to 0% in year 1990 and linear scale up until earliest measured data
Persistent diarrhea	None	Assumed coverage equal to 0% in 2000 and 33% in all countries

*DHS, MICS surveys and other similar surveys

Webappendix Table 2

Average coverage/prevalence of interventions in 50 countries included in analysis, weighted by number of live births in each country

Intervention	Data Assumptions	1980	2000	2015
Antibiotics for treatment of dysentery	1960 year of introduction and linear scale up until earliest measured data	12·33	18·69	21·06
Early Initiation of Breastfeeding	1980 coverage equal to earliest measured	26·03	37·52	57·97
Hand washing with soap	Equal to water connection in the home in 1980 and linear scale up to earliest measured data/ 2009 global estimate	8·62	13·88	18·29
Improved sanitation + improved water source	1960 year of introduction and linear scale up until earliest measured data	27·41	30·13	41·58
Rotavirus vaccine: two doses	Assumed 0 unless measured data available	0·00	0·00	25·47
ORS - oral rehydration solution	1980 year of introduction and linear scale up until earliest measured data	0·00	29·50	44·08
Persistent diarrhea treatment	0 in 2000 and linear scale up to 33% in 2015 in all countries	0·00	0·00	33·00
Vitamin A supplementation	0 in 1980· 1990 year of introduction and linear scale up until earliest measured data	0·00	26·07	68·54
Zinc for treatment of diarrhea	0 in 1980 and 0 until earliest measured data	0·00	2·41	12·02
Global stunting (<-2 SD) rate	Modeled stunting data used for 1980 (Majid et al)	57·30	48·67	36·55
Severe stunting rate (0-5 months)	Modeled stunting data used for 1980 (Majid et al)	9·73	11·21	8·68
Severe stunting rate (6-11 months)	Modeled stunting data used for 1980 (Majid et al)	14·10	16·08	12·04
Severe stunting rate (12-23 months)	Modeled stunting data used for 1980 (Majid et al)	29·34	33·37	24·71
Severe stunting rate (24-59 months)	Modeled stunting data used for 1980 (Majid et al)	35·34	38·74	28·53
Global wasting (<-2 SD) rate	1980 prevalence equal to earliest measured	14·99	12·91	13·29
Severe wasting (0-5 months)	1980 prevalence equal to earliest measured	8·58	10·12	11·62
Severe wasting (6-11 months)	1980 prevalence equal to earliest measured	8·04	9·12	10·23
Severe wasting (12-23 months)	1980 prevalence equal to earliest measured	7·03	7·28	8·57
Severe wasting (24-59 months)	1980 prevalence equal to earliest measured	3·88	3·87	5·91
Exclusive breastfeeding <1 month	1980 prevalence equal to earliest measured	56·68	61·80	63·17
Exclusive breastfeeding 1-5 months	1980 prevalence equal to earliest measured	35·79	37·24	40·62
Predominant breastfeeding <1 month	1980 prevalence equal to earliest measured	26·02	31·57	30·38
Predominant breastfeeding 1-5 months	1980 prevalence equal to earliest measured	25·52	37·68	37·69
Partial breastfeeding <1 month	1980 prevalence equal to earliest measured	14·60	13·24	16·14
Partial breastfeeding 1-5 months	1980 prevalence equal to earliest measured	35·29	38·49	38·97
Any breastfeeding 6-11 months	1980 prevalence equal to earliest measured	95·16	95·15	93·66
Any breastfeeding 12-24 months	1980 prevalence equal to earliest measured	76·71	79·13	74·87
No breastfeeding 0-1 month	1980 prevalence equal to earliest measured	3·31	3·37	3·82
No breastfeeding 1-5 months	1980 prevalence equal to earliest measured	4·05	2·74	4·18
No breastfeeding 6-11 months	1980 prevalence equal to earliest measured	5·43	6·68	9·21
No breastfeeding 12-24 months	1980 prevalence equal to earliest measured	23·88	26·64	34·59

Webappendix Table 3

Description of the interventions and risk factors included in each universal coverage scale-up scenario.

	Scenario 1<br>Direct Diarrhea Interventions	Scenario 2<br>Direct Diarrhea Interventions and Nutrition Interventions	Scenario 3<br>All interventions that affect diarrhea mortality
Interventions scaled up to universal coverage (90%)<br><br>(For stunting, target = reduction by 50%, for wasting target= <5%)	• Antibiotics for dystentery<br>• Oral rehydration solution<br>• Persistent diarrhea treatment<br>• Rotavirus vaccine<br>• Zinc	• All direct diarrhea interventions<br>• Vitamin A<br>• Stunting prevalence<br>• Wasting prevalence<br>• Breastfeeding practices<br>• Early initiation of breastfeeding	• All direct diarrhea interventions<br>• All nutrition interventions<br>• Improved sanitation + improved water source<br>• Handwashing

Webappendix Table 4

Number of diarrhea deaths among children under-five for 87 LMIC in 1980 and 2015

*VAMCM: Verbal-Autopsy-based Multi-Cause Modeling[7]

	1980			2015		
Rank	Country	MDG Region	Diarrhea Deaths	Country	MDG Region	Diarrhea Deaths
1	India	Southern Asia	844436	India	Southern Asia	117285
2	China	Eastern Asia	171276	Nigeria	Sub-Saharan Africa	76980
3	Nigeria	Sub-Saharan Africa	156788	Pakistan	Southern Asia	39484
4	Bangladesh	Southern Asia	150246	Democratic Republic of the Congo	Sub-Saharan Africa	32047
5	Indonesia	South-eastern Asia	119114	Angola	Sub-Saharan Africa	24927
6	Ethiopia	Sub-Saharan Africa	84475	Ethiopia	Sub-Saharan Africa	15535
7	Pakistan	Southern Asia	79648	Afghanistan	Southern Asia	11723
8	Democratic Republic of the Congo	Sub-Saharan Africa	55291	Chad	Sub-Saharan Africa	11360
9	Brazil	Latin America and the Caribbean	50216	Niger	Sub-Saharan Africa	9873
10	Egypt	Northern Africa	48334	Sudan	Sub-Saharan Africa	9536
11	Afghanistan	Southern Asia	37555	Somalia	Sub-Saharan Africa	8759
12	Cambodia	South-eastern Asia	36283	Indonesia	South-eastern Asia	8551
13	Myanmar	South-eastern Asia	33238	Cameroon	Sub-Saharan Africa	8032
14	Mozambique	Sub-Saharan Africa	32387	United Republic of Tanzania	Sub-Saharan Africa	8000
15	United Republic of Tanzania	Sub-Saharan Africa	30521	Mali	Sub-Saharan Africa	7807
16	Nepal	Southern Asia	29845	Mozambique	Sub-Saharan Africa	7341
17	Angola	Sub-Saharan Africa	25914	Bangladesh	Southern Asia	7189
18	Mali	Sub-Saharan Africa	25269	Uganda	Sub-Saharan Africa	7001
19	Niger	Sub-Saharan Africa	25145	Cote d'Ivoire	Sub-Saharan Africa	5697
20	Iran (Islamic Republic of)	Southern Asia	24844	Kenya	Sub-Saharan Africa	5442
21	Uganda	Sub-Saharan Africa	23408	China	Eastern Asia	5291
22	Malawi	Sub-Saharan Africa	20209	Philippines	South-eastern Asia	5075
23	Mexico	Latin America and the Caribbean	20029	Burkina Faso	Sub-Saharan Africa	4975
24	Philippines	South-eastern Asia	19294	Benin	Sub-Saharan Africa	3996
25	Burkina Faso	Sub-Saharan Africa	18023	Burundi	Sub-Saharan Africa	3716
26	Yemen	Western Asia	16989	Zimbabwe	Sub-Saharan Africa	3677
27	Sudan	Sub-Saharan Africa	16642	Madagascar	Sub-Saharan Africa	3665
28	Algeria	Northern Africa	16423	Ghana	Sub-Saharan Africa	3657
29	Morocco	Northern Africa	15467	South Africa	Sub-Saharan Africa	3627
30	Madagascar	Sub-Saharan Africa	15175	Guinea	Sub-Saharan Africa	3506
31	Kenya	Sub-Saharan Africa	14906	Zambia	Sub-Saharan Africa	3469
32	Peru	Latin America and the Caribbean	14551	Myanmar	South-eastern Asia	3273

33	South Sudan	Sub-Saharan Africa	14273	Egypt	Northern Africa	3271
34	South Africa	Sub-Saharan Africa	14082	South Sudan	Sub-Saharan Africa	3243
35	Guinea	Sub-Saharan Africa	13593	Malawi	Sub-Saharan Africa	3062
36	Cameroon	Sub-Saharan Africa	13398	Sierra Leone	Sub-Saharan Africa	2784
37	Somalia	Sub-Saharan Africa	13198	Yemen	Western Asia	2562
38	Ghana	Sub-Saharan Africa	12716	Senegal	Sub-Saharan Africa	2403
39	Cote d'Ivoire	Sub-Saharan Africa	12201	Viet Nam	South-eastern Asia	2261
40	Viet Nam	South-eastern Asia	11747	Central African Republic	Sub-Saharan Africa	2194
41	Rwanda	Sub-Saharan Africa	11212	Iraq	Western Asia	2119
42	Senegal	Sub-Saharan Africa	10983	Haiti	Latin America and the Caribbean	1860
43	Chad	Sub-Saharan Africa	10753	Togo	Sub-Saharan Africa	1636
44	Haiti	Latin America and the Caribbean	9525	Uzbekistan	Caucasus and Central Asia	1546
45	Zambia	Sub-Saharan Africa	9203	Brazil	Latin America and the Caribbean	1318
46	Bolivia (Plurinational State of)	Latin America and the Caribbean	8858	Lao People's Democratic Republic	South-eastern Asia	1298
47	Sierra Leone	Sub-Saharan Africa	8730	Algeria	Northern Africa	1218
48	Burundi	Sub-Saharan Africa	8502	Mauritania	Sub-Saharan Africa	1186
49	Benin	Sub-Saharan Africa	7892	Nepal	Southern Asia	1173
50	Guatemala	Latin America and the Caribbean	7464	Rwanda	Sub-Saharan Africa	1033
51	Uzbekistan	Caucasus and Central Asia	6823	Liberia	Sub-Saharan Africa	937
52	Zimbabwe	Sub-Saharan Africa	5808	Guatemala	Latin America and the Caribbean	931
53	Lao People's Democratic Republic	South-eastern Asia	5407	Mexico	Latin America and the Caribbean	920
54	Democratic People's Republic of Korea	Eastern Asia	5256	Tajikistan	Caucasus and Central Asia	918
55	Liberia	Sub-Saharan Africa	4873	Morocco	Northern Africa	906
56	Togo	Sub-Saharan Africa	4232	Iran (Islamic Republic of)	Southern Asia	892
57	Tajikistan	Caucasus and Central Asia	4119	Papua New Guinea	Oceania	810
58	Eritrea	Sub-Saharan Africa	4059	Eritrea	Sub-Saharan Africa	694
59	Iraq	Western Asia	4020	Cambodia	South-eastern Asia	682
60	Kazakhstan	Caucasus and Central Asia	3923	Bolivia (Plurinational State of)	Latin America and the Caribbean	579
61	Central African Republic	Sub-Saharan Africa	3595	Democratic People's Republic of Korea	Eastern Asia	563
62	Azerbaijan	Caucasus and Central Asia	2959	Guinea-Bissau	Sub-Saharan Africa	562
63	Timor-Leste	South-eastern Asia	2689	Lesotho	Sub-Saharan Africa	541
64	Dominican Republic	Latin America and the Caribbean	2545	Congo	Sub-Saharan Africa	519
65	Papua New Guinea	Oceania	2527	Peru	Latin America and the Caribbean	514
66	Mongolia	Eastern Asia	2007	Gambia	Sub-Saharan Africa	487

67	Turkmenistan	Caucasus and Central Asia	1964	Turkmenistan	Caucasus and Central Asia	484
68	Guinea-Bissau	Sub-Saharan Africa	1823	Azerbaijan	Caucasus and Central Asia	423
69	Mauritania	Sub-Saharan Africa	1738	Kazakhstan	Caucasus and Central Asia	318
70	Congo	Sub-Saharan Africa	1661	Namibia	Sub-Saharan Africa	299
71	Kyrgyzstan	Caucasus and Central Asia	1616	Timor-Leste	South-eastern Asia	253
72	Lesotho	Sub-Saharan Africa	1343	Dominican Republic	Latin America and the Caribbean	231
73	Gambia	Sub-Saharan Africa	1251	Swaziland	Sub-Saharan Africa	230
74	Bhutan	Southern Asia	899	Equatorial Guinea	Sub-Saharan Africa	200
75	Namibia	Sub-Saharan Africa	827	Kyrgyzstan	Caucasus and Central Asia	186
76	Swaziland	Sub-Saharan Africa	800	Gabon	Sub-Saharan Africa	160
77	Gabon	Sub-Saharan Africa	655	Botswana	Sub-Saharan Africa	157
78	Botswana	Sub-Saharan Africa	553	Comoros	Sub-Saharan Africa	142
79	Equatorial Guinea	Sub-Saharan Africa	475	Djibouti	Sub-Saharan Africa	116
80	Comoros	Sub-Saharan Africa	423	Mongolia	Eastern Asia	98
81	Djibouti	Sub-Saharan Africa	332	Solomon Islands	Oceania	34
82	Solomon Islands	Oceania	185	Bhutan	Southern Asia	26
83	Sao Tome and Principe	Sub-Saharan Africa	58	Sao Tome and Principe	Sub-Saharan Africa	23
84	Kiribati	Oceania	44	Kiribati	Oceania	15
85	Micronesia (Federated States of)	Oceania	19	Micronesia (Federated States of)	Oceania	5
86	Marshall Islands	Oceania	11	Marshall Islands	Oceania	4
87	Nauru	Oceania	1	Nauru	Oceania	0

Webappendix Table 5

Comparison of change in DSMR calculated from mortality trends versus trends that calculated from *LiST* projections in 50 LMIC

Country	Modeled in *LiST*			Mortality Trends			Percent of Reduction Explained by *LiST*	Most Recent Survey
	1980 DSMR	Most recent survey year DSMR	Percent change 1980 to most recent survey year	1980 DSMR	Most Recent Survey Year DSMR	Percent change 1980 to most recent survey yea		
Overall	34·9	15·7	55·5	35·5	5·7	79·9	69·5	
Afghanistan	54·1	29·2	46·1	58·3	11·5	80·3	57·4	DHS 2015
Angola	57·7	33·2	42·4	59·4	22·8	61·5	68·9	MICS 2001 + DHS 2015
Bangladesh	43·3	7·6	82·5	42·8	2·3	94·7	87·1	DHS 2014
Benin	44·2	32·5	26·5	45·4	11·3	75·1	35·3	MICS 2014
Burkina Faso	52·2	31·1	40·4	54·4	7·9	85·5	47·3	DHS 2010
Burundi	39·0	23·0	41·0	41·0	11·5	71·9	57·0	DHS 2010
Cameroon	32·4	19·3	40·2	33·2	10·5	68·3	58·9	MICS 2014
Central African Republic	37·2	25·6	31·0	37·6	16·0	57·5	53·9	MICS 2010
Comoros	29·5	22·2	24·6	30·1	6·8	77·3	31·8	DHS 2012
Congo	21·9	12·0	45·3	22·1	3·4	84·4	53·7	DHS 2014
Côte d'Ivoire	31·0	27·5	11·0	31·1	7·9	74·5	14·8	DHS 2011
Democratic Republic of the Congo	44·1	14·4	67·4	45·6	10·9	76·2	88·5	DHS 2013
Djibouti	27·0	17·3	35·9	24·6	8·3	66·4	54·1	DHS 2006
Egypt	28·6	14·9	48·1	28·9	1·2	95·7	50·3	DHS 2014
Ethiopia	47·1	24·4	48·2	48·6	7·1	85·4	56·4	DHS 2011
Gabon	23·2	15·9	31·6	23·9	4·0	83·3	37·9	DHS 2012
Gambia	38·6	20·1	48·0	40·8	7·6	81·4	59·0	DHS 2013
Ghana	26·8	8·7	67·5	27·0	4·3	84·0	80·4	DHS 2014
Guatemala	24·2	10·1	58·4	24·4	2·3	90·8	64·3	DHS 2014
Guinea-Bissau	44·3	13·2	70·2	46·8	9·4	79·9	87·9	DHS 2014
Haiti	39·6	18·4	53·6	41·0	8·7	78·8	68·0	DHS 2012
India	33·2	14·2	57·3	33·9	4·7	86·2	66·5	NFHS 2015
Iraq	7·6	5·2	31·9	7·5	2·2	71·0	44·9	MICS 2011
Kenya	18·6	5·9	68·1	18·9	3·9	79·5	85·7	DHS 2014
Kyrgyzstan	14·9	7·8	47·5	15·4	1·2	92·3	51·5	MICS 2014
Lao People's Democratic Republic	38·5	18·5	51·8	38·7	9·1	76·5	67·7	MICS 2011
Lesotho	32·0	11·6	63·8	25·4	9·1	64·4	99·1	DHS 2014
Liberia	51·9	18·8	63·8	53·5	7·1	86·7	73·6	DHS 2013
Malawi	60·2	14·9	75·3	62·6	4·9	92·2	81·7	DHS 2015
Mali	68·9	36·1	47·6	72·2	10·8	85·1	55·9	MICS 2015
Mauritania	26·2	9·8	62·5	26·6	9·0	66·1	94·6	MICS 2015
Mozambique	56·4	25·7	54·5	58·0	8·9	84·7	64·3	DHS 2011
Nepal	47·3	24·1	49·1	48·4	2·3	95·2	51·6	MICS 2014
Niger	73·1	48·8	33·2	77·4	13·6	82·4	40·3	DHS 2012

Nigeria	44·8	30·8	31·3	46·3	12·6	72·7	43·1	DHS 2013
Pakistan	24·3	12·5	48·6	24·4	8·2	66·5	73·1	DHS 2012
Peru	23·7	10·6	55·4	23·3	0·8	96·4	57·5	DHS 2014
Rwanda	40·5	12·8	68·4	41·5	3·4	91·7	74·6	DHS 2014
São Tomé and Príncipe	17·3	4·2	75·8	15·3	3·9	74·8	101·3	MICS 2014
Senegal	39·7	17·7	55·4	41·0	4·1	89·9	61·6	DHS 2015
Sierra Leone	59·1	16·1	72·8	61·4	15·4	74·9	97·2	DHS 2013
Swaziland	27·3	7·1	74·1	28·0	6·7	76·0	97·5	MICS 2014
Tajikistan	25·8	10·2	60·7	27·0	3·9	85·4	71·1	DHS 2013
Togo	32·6	16·5	49·3	33·0	7·1	78·5	62·8	DHS 2013
Turkménistan	19·9	9·4	52·5	20·0	4·2	78·8	66·6	MICS 2015
Uganda	37·9	19·5	48·4	38·3	5·8	84·9	57·0	DHS 2011
United Republic of Tanzania	34·9	11·1	68·2	35·3	3·9	89·0	76·6	DHS 2015
Viet Nam	6·8	2·1	68·9	6·8	1·5	77·7	88·7	MICS 2014
Zambia	32·2	11·6	63·8	32·6	6·8	79·1	80·7	DHS 2013
Zimbabwe	17·4	8·7	50·0	17·3	6·7	61·1	81·8	DHS 2015

Webappendix Table 6

The impact on diarrhea-specific mortality (DSMR) if increased coverage of different packages of interventions is achieved by 2030.

	2015	2030					
		Scenario 1		Scenario 2		Scenario 3	
Country	DSMR	DSMR	Percent Reduction	DSMR	Percent Reduction	DSMR	Percent Reduction
Global	6·02	1·56	74·06	0·66	89·09%	0·43	92·84%
Afghanistan	11·18	2·74	75·48	1·03	90·81%	0·66	94·06%
Angola	23·13	5·11	77·92	2·29	90·11	1·56	93·24
Bangladesh	2·30	0·98	57·52	0·45	80·31	0·32	86·00
Benin	10·47	2·55	75·64	0·90	91·44	0·56	94·66
Burkina Faso	7·14	1·60	77·53	0·61	91·41	0·38	94·64
Burundi	7·90	1·96	75·13	1·16	85·34	0·79	90·01
Cameroon	9·79	2·07	78·81	1·16	88·12	0·79	91·96
Central African Republic	14·08	2·72	80·67	1·20	91·49	0·75	94·68
Comoros	5·56	1·24	77·78	0·49	91·11	0·32	94·29
Congo	3·54	0·81	77·03	0·43	87·76	0·27	92·38
Côte d'Ivoire	6·94	1·38	80·11	0·73	89·42	0·46	93·36
DRC	10·20	2·40	76·43	1·17	88·55	0·75	92·68
Djibouti	5·38	1·72	68·13	0·39	92·71	0·25	95·45
Egypt	1·22	0·22	81·64	0·10	91·91	0·08	93·15
Ethiopia	4·89	1·17	76·12	0·57	88·27	0·37	92·51
Gabon	3·05	0·62	79·59	0·31	89·80	0·21	93·20
Gambia	6·09	1·81	70·20	0·76	87·54	0·54	91·15
Ghana	4·14	1·13	72·65	0·67	83·83	0·39	90·48
Guatemala	2·10	0·58	72·45	0·34	83·86	0·26	87·81
Guinea-Bissau	9·20	2·12	77·01	1·35	85·37	0·84	90·84
Haiti	7·05	1·83	73·97	1·05	85·08	0·67	90·52
India	4·66	1·26	73·04	0·41	91·22	0·27	94·18
Iraq	1·73	0·50	70·97	0·27	84·43	0·17	90·21
Kenya	3·46	0·94	72·91	0·54	84·47	0·36	89·69
Kyrgyzstan	1·12	0·26	76·44	0·16	85·64	0·13	88·38

Laos	7·48	1·75	76·64	0·98	86·96	0·72	90·40
Lesotho	6·91	1·82	73·70	1·13	83·69	0·72	89·53
Liberia	6·13	1·73	71·69	0·94	84·73	0·58	90·56
Malawi	4·29	1·50	64·95	1·18	72·56	0·77	81·97
Mali	10·62	2·39	77·53	1·30	87·81	0·82	92·30
Mauritania	9·00	2·34	73·97	0·95	89·49	0·63	93·01
Mozambique	6·19	1·67	73·09	0·88	85·79	0·55	91·13
Nepal	2·10	0·52	75·38	0·26	87·50	0·18	91·64
Niger	10·29	2·52	75·47	0·90	91·25	0·54	94·70
Nigeria	11·22	2·44	78·22	0·79	92·96	0·51	95·49
Pakistan	7·44	1·74	76·57	0·78	89·57	0·56	92·51
Peru	0·83	0·21	75·13	0·13	83·78	0·11	87·24
Rwanda	2·71	0·61	77·31	0·44	83·74	0·32	88·33
São Tomé and Príncipe	3·63	0·88	75·65	0·56	84·65	0·33	90·77
Senegal	4·13	1·05	74·65	0·57	86·1	0·40	90·42
Sierra Leone	12·44	6·43	48·35	2·59	79·16	1·58	87·28
Swaziland	3·72	1·93	48·14	1·07	71·33	0·75	79·93
Tajikistan	3·52	1·13	67·93	0·49	86·16	0·37	89·50
Togo	6·42	1·35	78·95	0·78	87·89	0·47	92·69
Turkmenistan	4·18	1·12	73·27	0·69	83·60	0·41	90·28
Uganda	4·03	0·97	76·06	0·56	86·21	0·34	91·52
United Republic of Tanzania	3·59	0·97	73·04	0·59	83·45	0·36	89·95
Vietnam	1·42	0·30	78·67	0·17	87·79	0·13	90·72
Zambia	4·63	1·48	67·99	0·86	81·53	0·58	87·57
Zimbabwe	5·88	1·47	75·05	0·78	86·80	0·51	91·30

Webappendix Table 7

The percent of child lives saved in 2030 attributable to each intervention in the three scale-up scenarios

Intervention	Percent Attributable		
	Scenario 1	Scenario 2	Scenario 3
**Antibiotics for dysentery**	7·3	3·2	2·0
**ORS**	51·6	22·8	14·4
**Persistent diarrhea treatment**	12·1	5.3	3·3
**Rotavirus vaccine**	12·0	8·6	6·8
**Zinc for treatment of diarrhea**	16·9	7·3	4·6
**Stunting prevalence**	0·00	21·6	17·1
**Wasting prevalence**	0·00	10·1	6·4
**Early initiation of breastfeeding**	0·00	0·15	0·1
**Age-appropriate breastfeeding practices**	0·00	17·3	13·5
**Vitamin A supplementation**	0·00	3·6	2·8
**Hand washing with soap**	0·00	0·00	15·7
**Improved sanitation and water**	0·00	0·00	13·1

Webappendix Table 8

Countries that would reduce diarrhea mortality to <1 death per 1000 live births in three scenarios

Direct Diarrhea	Direct Diarrhea Plus Nutrition	Direct Diarrhea Plus Nutrition and WASH	Unable to reduce DSMR to <1 in an scenario
Bangladesh	Benin	Afghanistan	Angola
Congo	Burkina Faso	Burundi	Sierra Leone
Egypt	Comoros	Cameroon	
Gabon	Côte d'Ivoire	Central African Republic	
Guatemala	Djibouti	Democratic Republic of the Congo	
Iraq	Ethiopia	Guinea-Bissau	
Kenya	Gambia	Haiti	
Kyrgyzstan	Ghana	Lesotho	
Nepal	India	Malawi	
Peru	Laos	Mali	
Rwanda	Liberia	Swaziland	
São Tomé and Principe	Mauritania		
Uganda	Mozambique		
United Republic of Tanzania	Niger		
Vietnam	Nigeria		
	Pakistan		
	Senegal		
	Tajikistan		
	Togo		
	Turkmenistan		
	Zambia		
	Zimbabwe		
